# Two-Dimensional Binary Superlattice of BNNT-Surfactant Vesicle Complex Induced by Electrostatic Interaction

**DOI:** 10.1021/acscentsci.5c00548

**Published:** 2025-05-22

**Authors:** Sang-Woo Jeon, Changwoo Do, Se Youn Moon, Tae-Hwan Kim

**Affiliations:** † Department of Applied Plasma & Quantum Beam Engineering, 26714 Jeonbuk National University, Jeonju 54896, Republic of Korea; ‡ Neutron Science Division, Korea Atomic Energy Research Institute, Daejeon 34057, Republic of Korea; § Biology and Soft Matter Division, Neutron Sciences Directorate, 6146 Oak Ridge National Laboratory, Oak Ridge, Tennessee 37831, United States; ∥ Research Center for Advanced Nuclear Interdisciplinary Technology, 26714 Jeonbuk National University, Jeonju 54896, Republic of Korea; ⊥ Department of Quantum System Engineering, 26714 Jeonbuk National University, Jeonju 54896, Republic of Korea; # High-Enthalphy Plasma Research Center, 26714 Jeonbuk National University, Wanju-gun 55317, Republic of Korea; ¶ Department of Electronic Engineering, 26714 Jeonbuk National University, Jeonju 54896, Republic of Korea; ■ Department of JBNU-KIST Convergence, 26714 Jeonbuk National University, Jeonju 54896, Republic of Korea

## Abstract

For a wide range of practical applications of boron nitride nanotubes (BNNTs), it is essential to achieve their highly ordered self-assembled structures. This study reports on a two-dimensional (2D) binary superlattice of individually exfoliated BNNTs with a negative surface charge (p-BNNT25) and cationic surfactant vesicles (CTAT/SDBS vesicles, prepared by mixing cetyltrimethylammonium tosylate (CTAT) and sodium dodecylbenzenesulfonate (SDBS)) complexes through electrostatic interactions. Depending on the surface charge density of the CTAT/SDBS vesicles and the mass ratio between the CTAT/SDBS vesicle and p-BNNT25, the CTAT/SDBS-BNNT complexes formed highly ordered superstructures. These structures include an intercalated lamellar phase with a centered rectangular structure (ICLP), in which a 2D array of p-BNNT25 is inserted into the multilamellar structure, and an AB_3_ structure, in which the BNNTs are surrounded by surfactant micelles in a triangular arrangement. To the best of our knowledge, this is the first demonstration of the fabrication of highly ordered superstructures of individually exfoliated and negatively charged BNNTs with positively charged surfactant vesicles through electrostatic interactions. This approach for the 2D binary superlattices of CTAT/SDBS-BNNT complexes induced by electrostatic interactions is expected to be beneficial for a wide range of one-dimensional (1D) nanoparticle applications.

## Graphical abstract

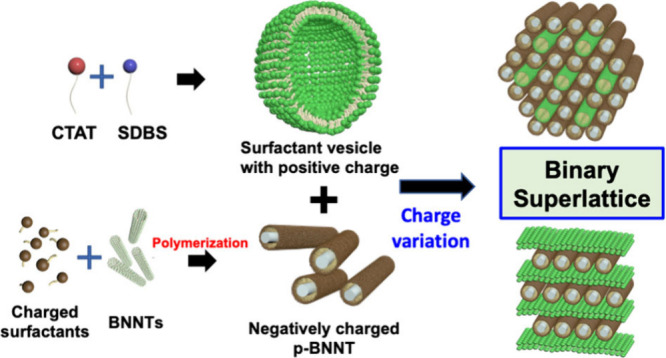

## Introduction

Boron nitride nanotubes (BNNTs) have attracted significant attention owing to their remarkable electrical,^1–4^ thermal,^5–8^ mechanical,^9–13^ and neutron shielding properties^14–16^ and have been used in a wide range of applications, including nanoscale piezoelectric devices,^4,16–19^ an electrical insulator with high thermal conductivity,^20–22^ reinforcement for materials,^23–26^ and neutron shielding materials.^16,18,27,28^ However, for practical applications of BNNTs, it is essential to fabricate nanostructures of individually exfoliated BNNTs with density and morphology in a specific region, that is, a highly ordered superlattice structure. The highly ordered structures of BNNTs are significant, because they facilitate the alignment of BNNTs, thereby enhancing heat dissipation and neutron shielding applications. Moreover, these ordered structures improve the physical properties of BNNTs, including their mechanical strength, thermal conductivity, and neutron shielding capabilities.^29–31^ Although there are a lot of reports on the fabrication of highly ordered superstructures of one-dimensional (1D) nanoparticles,^29,32–38^ achieving such structures by controlling external interactions requires complex processing and remains particularly challenging for BNNTs, which are still in the early stages. In fact, there is only one report, in which it was prepared by hydrophobic interactions of BNNTs with amphiphilic block copolymers;^29^ however, a simpler and more immediate method remains a challenge.

Charged nanoparticles readily self-assemble into a wide variety of highly ordered structures through electrostatic interactions. Many highly ordered structures have been successfully fabricated by electrostatic interactions, which are highly dependent on their shape or dimensionality.^38–50^ For 1D nanoparticles, in particular, it is difficult to achieve various highly ordered structures owing to the anisotropy of the nanoparticles. Nevertheless, the results for 1D nanoparticles have been reported through the electrostatic interaction between the nanoparticle and soft materials such as polymers and lipids (i.e., DNA-lipid complexes,^43–47^ SWNT-lipid or polymer complexes,^48,49^ and 1D virus-lipid complexes^50^). Considering that DNA, SWNT, and viruses are charged 1D nanoparticles, we expect that previous studies can provide a path for the fabrication of highly ordered superstructures of 1D nanoparticles by electrostatic interactions with surrounding materials. In this manner, the charged BNNTs, which are also 1D nanoparticles, can self-assemble into highly ordered superstructures, depending on electrostatic interactions.

This study demonstrates highly ordered superstructures of BNNTs fabricated by electrostatically interacting between negatively charged BNNTs (p-BNNT25s) (which are prepared by *in situ* free radical copolymerization of cetyltrimethylammonium 4-vinyl benzoate (CTVB) and anionic salt, sodium styrenesulfonate (NaSS), adsorbed on the BNNT surface)^51–53^ and cationic surfactant vesicles (which are prepared by mixing cetyltrimethylammonium tosylate and sodium dodecylbenzenesulfonate (CTAT/SDBS)) in aqueous solution.^54–56^ For utilizing the electrostatic interaction with cationic surfactant vesicles, the surfaces of BNNTs, which are electrically neutral, were modified into negatively charged surfaces through the copolymerization of CTVB and NaSS, where the surface charge of BNNT was controllable with the NaSS concentration (Figure S1). Depending on the charge interaction, an intercalated lamellar structure with a centered rectangular symmetry and a hexagonally packed BNNT structure with a triangular arrangement of cylindrical micelles surrounding the p-BNNT25 were observed. The charge interaction was controlled by the mixing ratio of p-BNNT25 and CTAT/SDBS vesicles (Figure 1). To the best of our knowledge, this is the first report of the fabrication of a self-assembled two-dimensional binary superlattice of BNNTs through electrostatic interactions, which provides a new way to fabricate collective BNNTs nanostructures with enhanced physical properties. This also opens up practical applications for surfactant-BNNT composites with multiple functionalities. Furthermore, it offers a cost-effective and simple method for the production of various highly ordered self-assemblies of individually exfoliated BNNTs.

**Figure 1:**
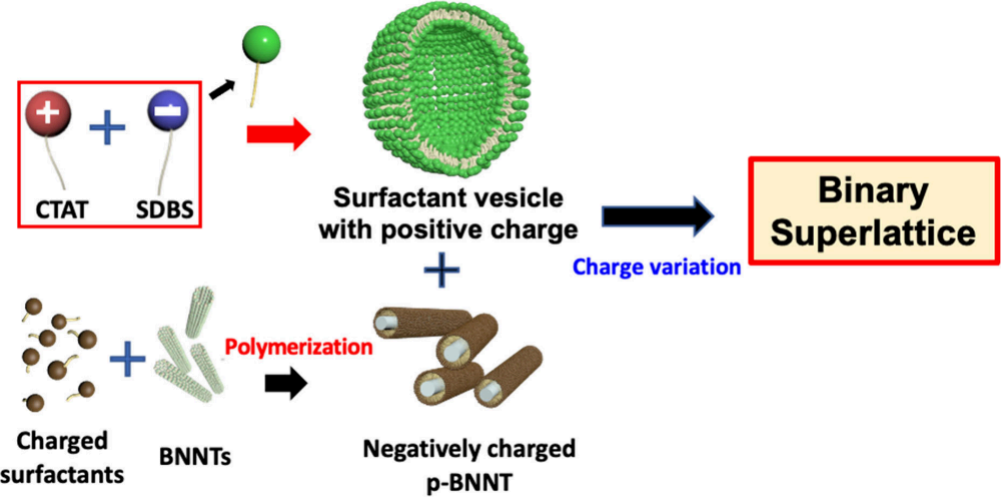
Schematics of the formation of a highly ordered superstructure of the CTAT/SDBS-BNNT complex by electrostatic interaction.

## Results and Discussion

Individually exfoliated and negatively charged BNNTs (p-BNNT25) were prepared by encapsulating CTVB and NaSS on the BNNT surface, followed by *in situ* free-radical copolymerization for permanent fixation, as confirmed by proton NMR measurements.^51–53^ The proton NMR spectra of the p-BNNT25 dispersion in D_2_O showed that the peaks of the vinyl groups (5.21, 5.73, and 6.61 ppm) and aromatic groups (7.33, 7.63, and 7.78 ppm) of the polymerizable counterions (VB and SS ions) were nearly absent compared with those of the unpolymerized BNNT dispersion in D_2_O (Figure S2). The significant disappearance of the peaks of VB and SS ions was caused by their reduced mobility after copolymerization and therefore exhibited a shortened T_2_-relaxation time, clearly indicating that all VB and SS ions have been entirely polymerized. The surface charge density of BNNT could be controlled by the NaSS concentration, which was confirmed by zeta potential measurements. In this study, the p-BNNT25 (which has 25 mol % NaSS concentration (relative to CTVB)) with −25.11 ± 2.77 mV of zeta potential was used as a representative surface charge. Detailed structural information on p-BNNT25 was obtained using SANS and AFM measurements. Through SANS model fitting using the sum of a core–shell cylinder (p-BNNT25) and a cylinder (p-CTVB) model, the cross-sectional distribution of p-BNNT25 (with a BNNT thickness of 3.0 ± 0.17 nm and a shell composed of surfactant molecules with a thickness of 1.6 ± 0.07 nm) was estimated (Figure 2a).^57–59^ The length information on p-BNNT25 was out of range in the SANS measurement (over hundreds of nanometers); however, the length of BNNT was obtained by analyzing the AFM images (in which the length distribution peak was 449.85 ± 35.71 nm). Furthermore, the diameter of BNNTs was also obtained from the AFM images (in which the diameter distribution peak was 3.01 ± 0.24 nm). Sectional information about the diameter and length in the AFM image was obtained using the Nano Scope Analysis Software provided by Bruker. Here, the length and diameter distribution peaks of BNNTs are the most probable values, which were estimated by a simple model fit with a log-normal distribution function (Figure 2b and 2c). The diameter of the BNNTs was consistent with that of the SANS analysis. The prepared p-BNNT25s were very stable and easily redispersed in aqueous solution, even after freeze-drying. To confirm the stability and redispersibility of p-BNNT25 after freeze-drying, the SAXS intensities and UV–vis absorbances of p-BNNT25 before and after freeze-drying were compared, which were identical, thereby indicating excellent redispersibility and structural stability of p-BNNT25 in water (Figure 2d and 2e).

**Figure 2:**
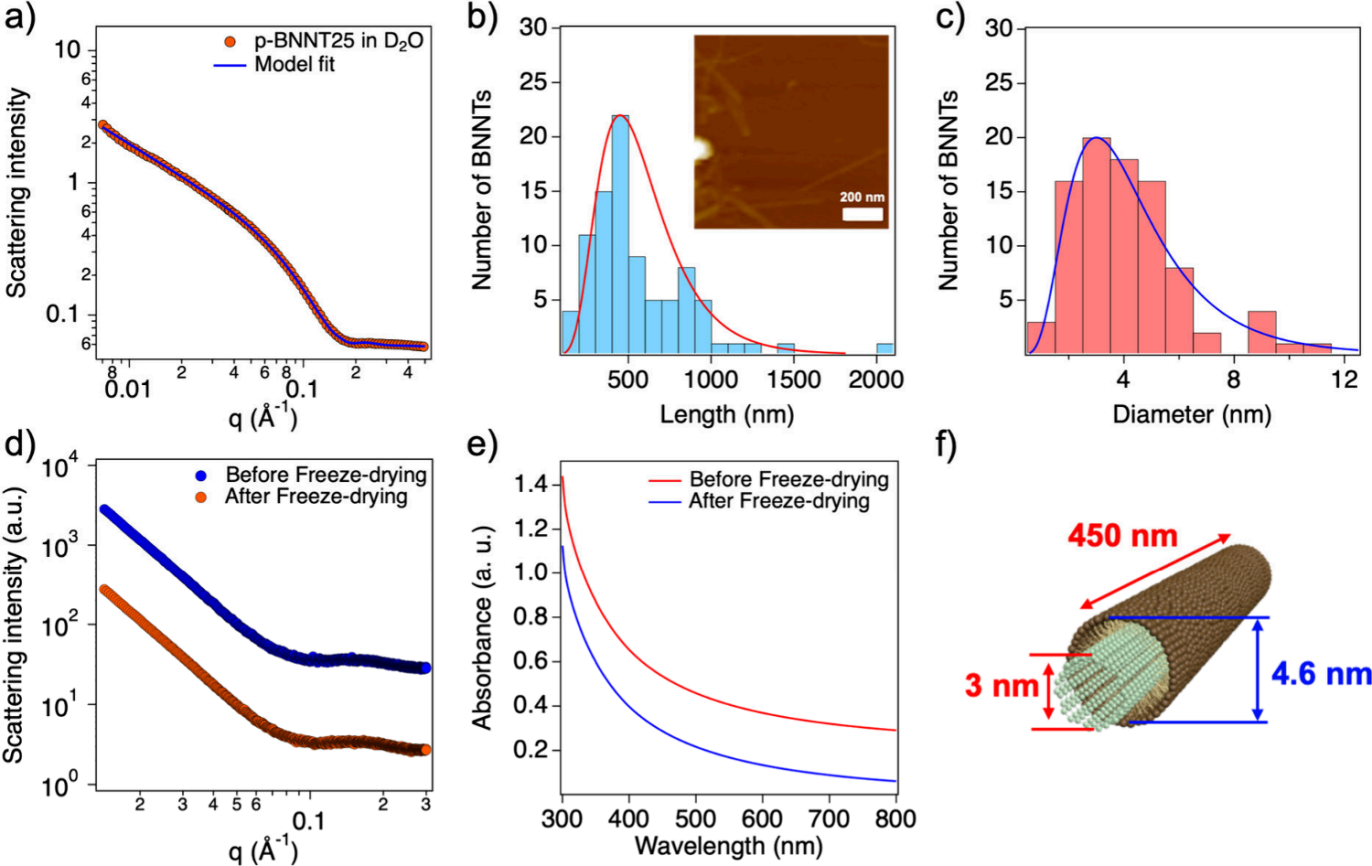
a) SANS form factor analysis of p-BNNT25 through model fitting. b) Length and c) diameter distribution of p-BNNT25 obtained from AFM analysis and its AFM image (inset in Figure 2b). Comparison of the d) SAXS intensities and e) UV–visible absorption spectra of p-BNNT25s before and after freeze-drying. f) Schematics of the structural information on p-BNNT25, as determined through SAXS and AFM analysis.

Highly ordered BNNT superstructures were fabricated through electrostatic interactions between negatively charged p-BNNT25 and cationic surfactant vesicles. To systematically control the electrostatic interactions, two types of oppositely charged surfactants of cetyltrimethylammonium tosylate (which is a cationic surfactant, CTAT) and sodium dodecylbenzenesulfonate (which is an anionic surfactant, SDBS) were used. The surface charge density of the CTAT/SDBS vesicles was modulated by mixing the vesicles with different mole fractions of the surfactants. It has been established that the mixture of CTAT and SDBS spontaneously forms vesicle structures in aqueous solution, depending on their total concentration and the mole fraction of CTAT (Φ_CTAT_).^54,55^ In this study, the total concentration of the CTAT/SDBS mixtures with different Φ_CTAT_ (= 0.733, 0.750, 0.765, and 0.779) was fixed at 1.25 wt %, and their structures were confirmed by SANS measurements (Figure S3a). Considering that interparticle interference was not observed and the SANS intensities exhibited *q* ^–2^ behavior, the structural information on the CTAT/SDBS mixtures was confirmed by a simple form factor analysis using a core–shell sphere with the scattering length density of the core equal to that of the solvent (vesicle) model.^56–58^ All of the SANS intensities were fully reproduced by a simple form factor with the core–shell spherical shape without a topological phase transition depending on Φ_CTAT_. When the Φ_CTAT_ increased from 0.733 to 0.779, the bilayer thickness and core radius of the surfactant vesicles slightly increased from 2.71 to 2.80 nm and from 25.12 to 25.78 nm, respectively (Figure S3b). The surface charge density of the CTAT/SDBS vesicles with different Φ_CTAT_ values was evaluated through zeta potential measurements. As the Φ_CTAT_ increased, the zeta potential of the CTAT/SDBS vesicles increased from +24.10 to +35.30 mV (Figure S4), which indicates a gradual increase in the surface charge density. Therefore, the electrostatic interactions between the CTAT/SDBS vesicles and p-BNNT25 can be easily controlled by adjusting the surface charge density of the CTAT/SDBS vesicles. Consequently, the electrostatic interactions between the CTAT/SDBS vesicles and p-BNNT25s can be controlled when the CTAT/SDBS vesicles and p-BNNT25s are mixed (that is, the CTAT/SDBS-BNNT complex), leading to the formation of various highly ordered superstructures of BNNTs in the CTAT/SDBS-BNNT complex that are entropically stabilized.

To prepare the CTAT/SDBS-BNNT complex with different electrostatic interactions between the surfactants and p-BNNT25, the CTAT/SDBS vesicles with different Φ_CTAT_ and p-BNNT25s were mixed at various mass ratios of CTAT/SDBS vesicles and p-BNNT25s (S/P), while the total concentration of p-BNNT25 and CTAT/SDBS vesicles was fixed at 1.2 wt %. The cooperative self-assembly of CTAT/SDBS-BNNT complexes, driven by the release of bound counterions as the p-BNNT25s and CTAT/SDBS vesicles compensate each other electrostatically,^45–47^ occurs most strongly at their electrically neutral point (that is, the isoelectric point), which was confirmed by zeta potential measurements of the complexes with different S/P (Figure S5). As the surface charge density of the CTAT/SDBS-BNNT complex increased (with increasing Φ_CTAT_ values (0.733, 0.750, 0.765, 0.779)), the S/P at the isoelectric point decreased from 1.0 to 0.6 (Figure 3), which was attributed to the increased need for p-BNNT25s to electrostatically compensate the CTAT/SDBS vesicles with a higher surface charge density. This indirectly supports the fact that the surface charge density of CTAT/SDBS vesicles was positively increased by Φ_CTAT_.

**Figure 3:**
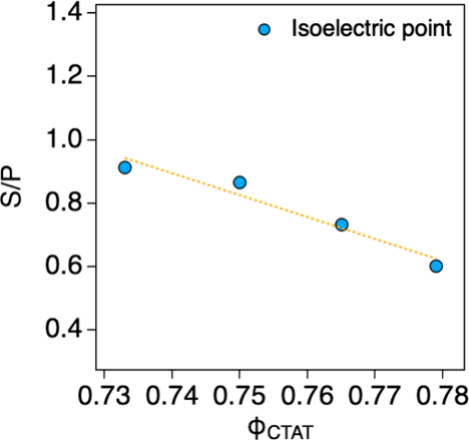
S/P at isoelectric points for CTAT/SDBS-BNNT complexes (Φ_CTAT_ = 0.733, 0.750, 0.765, and 0.779).

The self-assembled structures of the CTAT/SDBS-BNNT complexes at the isoelectric points (S/P = 1.0, 0.8, 0.7, and 0.6 for Φ_CTAT_ = 0.733, 0.750, 0.765, and 0.779, respectively) were analyzed by using the SAXS measurements obtained from the 4C beamline of the Pohang Accelerator Laboratory (PAL). All the SAXS intensities of the CTAT/SDBS-BNNT complexes at their isoelectric points revealed 12–13 strong and sharp Bragg peaks arising from the highly ordered cooperative self-assemblies of the complexes (Figure 4a). In this SAXS analysis, because many Bragg peaks were observed and not all peaks were successfully matched with a highly ordered structure in a single phase, they were classified into two sets of Bragg peaks to describe the complicated nanostructure of the CTAT/SDBS-BNNT complexes. First, the five sharp peaks (*q* = 0.067, 0.134, 0.200, 0.267, and 0.334 Å^–1^, indicated using black arrows) of the CTAT/SDBS-BNNT complex (Φ_CTAT_ = 0.733, S/P = 1.0) correspond to the (001), (002), (003), (004), and (005) Bragg reflections of the multilamellar structures with lamellar repeat distances (*d*_lam_ = 9.41 nm (= 2 π/*q*_(100)_), Figure 4b). The two broad peaks (indicated using red arrows) were also observed at approximately 0.130 and 0.160 Å^–1^, which can be indexed with the (1,1) and (1,3) Bragg reflections of a centered rectangular columnar packing of p-BNNT25s that occurred at qhk=2π(ha)2+(kb)2 , where, *a* and *b* are the two-dimensional lattice parameters of the centered rectangular structure.^60^ It should be noted that the lattice parameter *b* is exactly 2 times the lamellar repeat distance (*b* = 18.82 nm (= 2*d*_lam_), Figure 4b), and the (0,2), (0,4), (0,6), and (0,8) Bragg reflections of the centered rectangular structure completely overlap with those of the multilamellar structure (indicated using black arrows in Figure 4a). The lattice parameter *a* was estimated to be 5.01 nm, which is slightly larger than the diameter of the p-BNNT25. Furthermore, the water gap in the multilamellar structure (which is estimated by the difference between the lamellar repeat distance (∼9.41 nm) and the surfactant bilayer thickness (∼2.70 nm)) was approximately 6.69 nm and is sufficient to accommodate as a monolayer of p-BNNT25 (diameter = 4.60 nm). Therefore, the p-BNNT25 monolayer and lamellar bilayer were alternately stacked (that is, the intercalated lamellar structure) with centered rectangular packing of p-BNNT25 (ICLP, Figure 4c). This further supports the centered symmetry because the (*h,k*) Bragg peaks with *h* + *k* = 2*n* + 1 such as (0,3), (1,2), (0,5), (1,4), and (0,7), were systematically absent in the covered *q* range in our SAXS experiments.^48,49,60^

**Figure 4:**
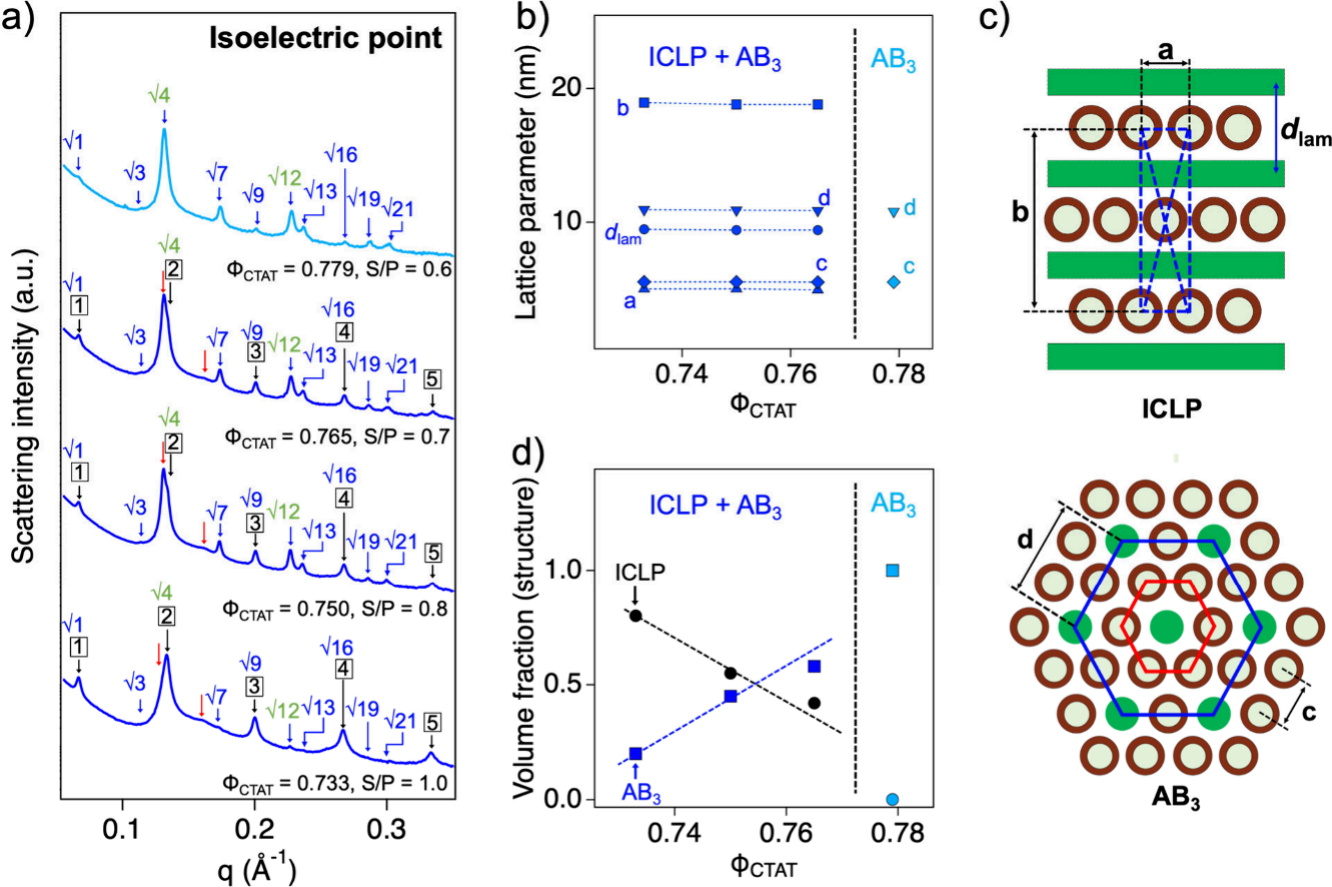
a) SAXS intensities of the CTAT/SDBS-BNNT complexes at the isoelectric point. Black, red, green and blue arrows represent the peaks from the ICLP and AB_3_ structures. b) Lattice parameter of the CTAT/SDBS-BNNT complex at the isoelectric point with different Φ_CTAT_. Illustration of the ICLP and AB_3_ structures of the CTAT/SDBS-BNNT complex at the isoelectric point. c) Illustration of the ICLP and AB_3_ structures of the CTAT/SDBS-BNNT complex at the isoelectric point. d) Calculated volume fraction of the ICLP and AB_3_ structures in the CTAT/SDBS-BNNT complexes at the isoelectric points with different Φ_CTAT_.

Second, ten additional Bragg peaks (*q* = 0.067, 0.115, 0.133, 0.173, 0.200, 0.231, 0.241, 0.267, 0.291, and 0.306 Å^–1^, indicated using blue arrows) were successfully matched with a two-dimensional (2D) hexagonal columnar structure (nearest neighboring particle–particle distance, *d* = 10.86 nm), which had a 1:√3:2:√7:3:√12:√13:√16:√19:√21 ratio of peak positions (Figure 4a). Here, the first, third, fifth, and eighth peaks overlapped with the Bragg reflections arising from the multilamellar structures. It should be noted that the peak intensities of the third (0.133 Å^–1^) and sixth peaks (0.231 Å^–1^) were rather strong (indicated using green), even though they were higher-order Bragg peaks. Furthermore, considering that the ratio of the *q* positions was 1:√3 and the nearest neighboring particle–particle distance of the 2D hexagonal columnar structure (lattice parameter *c*) was 5.43 nm, which is half of *d*, it is possible that the CTAT/SDBS-BNNT complex formed two different sets of hexagonal structures with *d* = 2*c* (Figure 4b). Because the CTAT/SDBS-BNNT complexes were formed by self-assembly driven by electrostatic interactions between oppositely charged surfactant vesicles and p-BNNT25, they could be formed into a cooperative structure of p-BNNT25s and surfactants, which is the AB_3_ structure, where three p-BNNT25s surround a single surfactant micelle (Figure 4c).^61^ In fact, when the electrostatic interaction becomes strong, the lattice parameters of the p-BNNT25s should decrease because a greater number of p-BNNT25s are required to compensate for the electrostatic interactions. Despite the increase of the electrostatic interaction with the increase of Φ_CTAT_, the lattice parameters of the CTAT/SDBS-BNNT complexes at the isoelectric point were nearly constant. Considering the structure of p-BNNT25s (diameter = 4.61 nm), even though it is easier to form an ICLP structure rather than an AB_3_ structure after electrostatic interaction (because it is hard to rupture the membrane bilayer), the CTAT/SDBS-BNNT complexes above a certain specific Φ_CTAT_ value cannot form the ICLP structure alone because the p-BNNT25s in the ICLP complex should not be sterically overlapped. Therefore, a topologically different structure is required to compensate for the electrostatic interaction of the CTAT/SDBS-BNNT complexes, leading to the additional formation of an AB_3_ structure with more p-BNNT25s.

As previously confirmed, when Φ_CTAT_ increased to 0.779, the S/P at the isoelectric point decreased from 1.0 to 0.6, which necessitated more p-BNNT25s to compensate the electrostatic interaction. It should be noticeable that the Bragg reflection corresponding to the AB_3_ structure became strong when the S/P decreased (the Φ_CTAT_ increased), which indicates the increase of the relative fraction of the AB_3_ structure in the CTAT/SDBS-BNNT complexes. At Φ_CTAT_ = 0.779, Bragg reflections of the ICLP structure completely disappeared, which indicates that only the AB_3_ structure existed in the CTAT/SDBS-BNNT complex, in which the lattice parameters did not change. Considering that the surface charge density of CTAT/SDBS vesicle becomes strong by increasing the CTAT concentration, it is natural to have strong electrostatic interaction in the CTAT/SDBS-BNNT complex. Since the surface charge density of p-BNNT25 is constant, however, we expect that the cationic charge of lamellar structure can be completely compensated with p-BNNT25 due to the steric hindrance when the CTAT/SDBS-BNNT complex forms to ICLP structure. Therefore, the surfactant bilayer of the surfactant vesicle was ruptured and reassembled into cylindrical micelles of AB_3_ structure to compensate the electrostatic interaction. The increase in the relative fraction of the AB_3_ structure in the CTAT/SDBS-BNNT complexes was confirmed by estimating the volume fractions of the ICLP and AB_3_ structures based on their geometrical structures and composition ratios (S/P). The volume fractions of each structure were calculated from the volume ratios (V_s_/V_p_) of the surfactants and p-BNNT25s in the ICLP and AB_3_ structures, which were obtained from the geometry of the structure determined by SANS analysis. As the lattice parameters of each structure were almost constant, the estimated V_s_/V_p_ values of the ICLP and AB_3_ structures did not change (0.818 and 0.252, respectively). The calculated volume fraction of the AB_3_ structures in the CTAT/SDBS-BNNT complexes increased from 20.20 to 57.10% with increasing Φ_CTAT_ (from 0.733 to 0.765) (Figure 4d). In contrast, the volume fraction of the ICLP structure, which had a relatively high V_s_/V_p_ compared to that of the AB_3_ structure, decreased from 79.80% to 42.10%. This strongly supports the hypothesis that the relative fraction of the AB_3_ structure in the CTAT/SDBS-BNNT complexes increases with Φ_CTAT_.

To support the SAXS analysis, transmission electron microscopy (TEM) measurements were performed on both the CTAT/SDBS (Φ_CTAT_ = 0.750) mixture and CTAT/SDBS-BNNT (Φ_CTAT_ = 0.750, S/P = 1.0) complex (Figure S6a-c). The TEM image of the CTAT/SDBS-BNNT complex revealed ordered structures with a *d*-spacing of approximately 5 nm, while the TEM image of the CTAT/SDBS mixture showed only vesicular structures with a diameter of approximately 50 nm (which is consistent with the result of SANS analysis (Figure S3a)). Considering that the lattice parameter *c* of the AB_3_ structure was 5.43 nm and the sample was dried, the ordered structures observed in the TEM image were comparable to the SAXS analysis. In addition, the energy dispersive spectroscopy (EDS) mapping images show that the ordered structure is composed of nitrogen (N) and boron (B) elements (Figure S6d and S6e), which means that the ordered structure in the TEM image is formed by p-BNNT25.

It is important to control the electrostatic interactions of the CTAT/SDBS-BNNT complexes to fabricate various highly ordered structures of BNNTs. To expand the scope of the electrostatic interactions in the CTAT/SDBS-BNNT complex, a CTAT/SDBS-BNNT complex with an S/P of nonisoelectric points was prepared. As the CTAT/SDBS-BNNT complex moves away from the isoelectric point by varying S/P, the electrostatic interactions between the CTAT/SDBS vesicles and p-BNNT25s become weaker because of the unbalanced charge distribution in the complex. Here, the CTAT/SDBS-BNNT complexes were prepared in the S/P range of 0.6 ∼ 1.8, which are nonisoelectric points.

The structures of the CTAT/SDBS-BNNT complexes, including the nonisoelectric points, were also analyzed using the 4C SAXS beamline of the Pohang Accelerator Laboratory (PAL) (Figure 5). In the CTAT/SDBS-BNNT complex with Φ_CTAT_ = 0.733, when the S/P increased from 0.6 to 0.9, which is less than the S/P at the isoelectric point, the SAXS intensities revealed five sharp peaks (*q* = 0.067, 0.134, 0.200, 0.267, and 0.334 Å^–1^, indicated using black arrows) corresponding to the Bragg’s reflections (001), (002), (003), (004), and (005) multilamellar structures. Furthermore, two broad peaks (indicated using red arrows) corresponding to the (1,1) and (1,3) Bragg reflections of a centered rectangular columnar packing of p-BNNT25s were also observed (Figure 5a). The lattice parameters are shown in Figure 6a. As described for the CTAT/SDBS-BNNT complex at the isoelectric point, these seven scattering peaks indicate the ICLP structure. Interestingly, the CTAT/SDBS-BNNT complexes at the nonisoelectric point formed an ICLP structure, even though the S/P was rather small. This can be understood as a weak electrostatic interaction because the CTAT/SDBS-BNNT complexes were prepared at nonisoelectric points and the surface charge density of the CTAT/SDBS vesicles was low. When the S/P increased above the isoelectric point (S/P = 1.4 and 1.8), two additional peaks were still observed, which corresponded to the fourth and sixth peaks of the AB_3_ structure, indicating the formation of multiphase, ICLP, and AB_3_ structures as a result of the isoelectric point. This can be explained by the fact that as the CTAT/SDBS-BNNT complex moves far away from the isoelectric point, an inhomogeneous charge distribution occurs within the complex, leading to unexpected electrostatic interactions and resulting in the partial formation of the AB_3_ structure.

**Figure 5:**
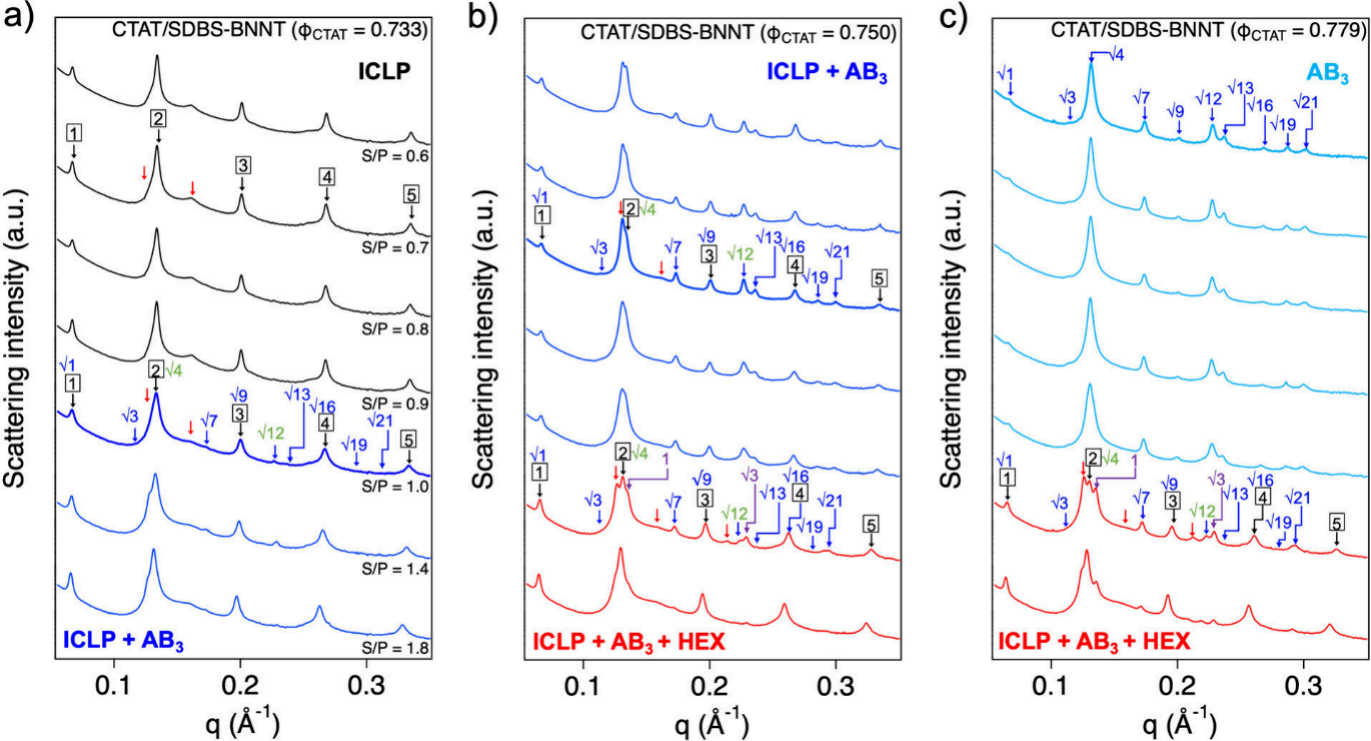
SAXS intensities of the CTAT/SDBS-BNNT composites with Φ_CTAT_ of a) 0.733, b) 0.750, and c) 0.779 at isoelectric and nonisoelectric points. Black, red, blue, green and purple arrows represent peaks from the ICLP, AB_3_, and HEX structures.

**Figure 6:**
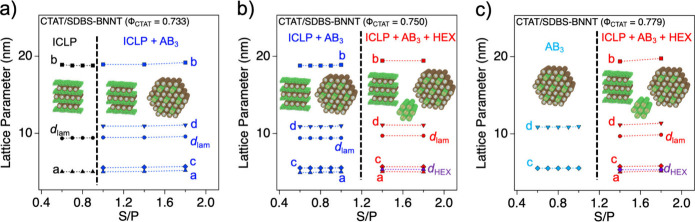
Lattice parameters (nm) of the CTAT/SDBS-BNNT composites with Φ_CTAT_: a) 0.733, b) 0.750, and c) 0.779.

In the CTAT/SDBS-BNNT complex with Φ_CTAT_ = 0.750 and 0.765 at nonisoelectric points (S/P = 0.6 ∼ 1.0), the SAXS intensities exhibited 12–13 strong and sharp Bragg’s peaks arising from the multiphase of the ICLP and AB_3_ structures as the results of the complex at the isoelectric points (Figure 5b and S7a). As the S/P further moved away from the isoelectric point (S/P = 1.4 and 1.8), two new peaks (indicated by purple arrows) appeared at *q* ∼ 0.136 Å^–1^ and ∼ 0.230 Å^–1^, which had a 1:√3 ratio of the peak positions, indicating the formation of a new hexagonal structure with a different lattice parameter (*d*_HEX_). Considering that the CTAT/SDBS-BNNT complexes at the nonisoelectric points (S/P = 1.4 and 1.8) have relatively high surfactants, it is presumed that the new hexagonal structure (HEX) consists of only surfactants. Therefore, the CTAT/SDBS-BNNT complexes with Φ_CTAT_ = 0.750 and 0.765 at nonisoelectric points (S/P = 1.4 and 1.8) comprised the ICLP, AB_3_, and HEX structures. All of the lattice parameters are shown in Figures 6b and S7b.

In the CTAT/SDBS-BNNT complex with Φ_CTAT_ = 0.779 at nonisoelectric points (S/P = 0.7 ∼ 1.0), the SAXS intensities exhibited 10 strong and sharp Bragg peaks arising from the AB_3_ structures, which was similar to the results of the complex at the isoelectric points (Figure 5c). As the S/P further moved away from the isoelectric point (S/P = 1.4 and 1.8), the two additional peaks (indicated by purple arrows) were also observed at *q* ∼ 0.136 Å^–1^ and ∼ 0.230 Å^–1^, which indicates the formation of a new hexagonal structure with a different *d*_HEX_. The lattice parameters are listed in Figure 6c. This was identical to the result of the CTAT/SDBS-BNNT complex with Φ_CTAT_ = 0.750 and 0.765. Therefore, the phase transition (AB_3_ – AB_3_ + HEX) of the CTAT/SDBS-BNNT complexes at nonisoelectric points can be explained in the manner of their composition ratio.

The SAXS intensities of CTAT/SDBS-BNNT (Φ_CTAT_ = 0.733, S/P = 0.7) and (Φ_CTAT_ = 0.779, S/P = 0.6), which exhibit the ICLP and AB_3_ structures, were compared with the simulated SAXS pattern by using the Powder Cell (v2.4) program. Considering the composition and symmetry of ICLP and AB_3_ structures, the experimental SAXS intensities of ICLP and AB_3_ structures are comparable to the simulated intensities based on the space group *C*2/*c* (66) (which has a centered rectangular symmetry) and *P*6/*mmm* (191) (which has a hexagonal symmetry), respectively (Figure S8).

Consequently, highly ordered superstructures of BNNTs were successfully fabricated by electrostatic interactions at isoelectric and nonisoelectric points, which were controlled by the surface charge density of the CTAT/SDBS vesicles and the mass ratio (S/P) between the CTAT/SDBS vesicles and p-BNNT25s. Based on intensive SAXS analysis, it was confirmed that the CTAT/SDBS-BNNT complexes self-assembled into two-dimensional binary superlattice structures (ICLP and AB_3_) with a HEX structure, depending on the electrostatic interactions and the composition of the complex. The phase behaviors of the CTAT/SDBS-BNNT complexes are summarized in Figure 7.

**Figure 7:**
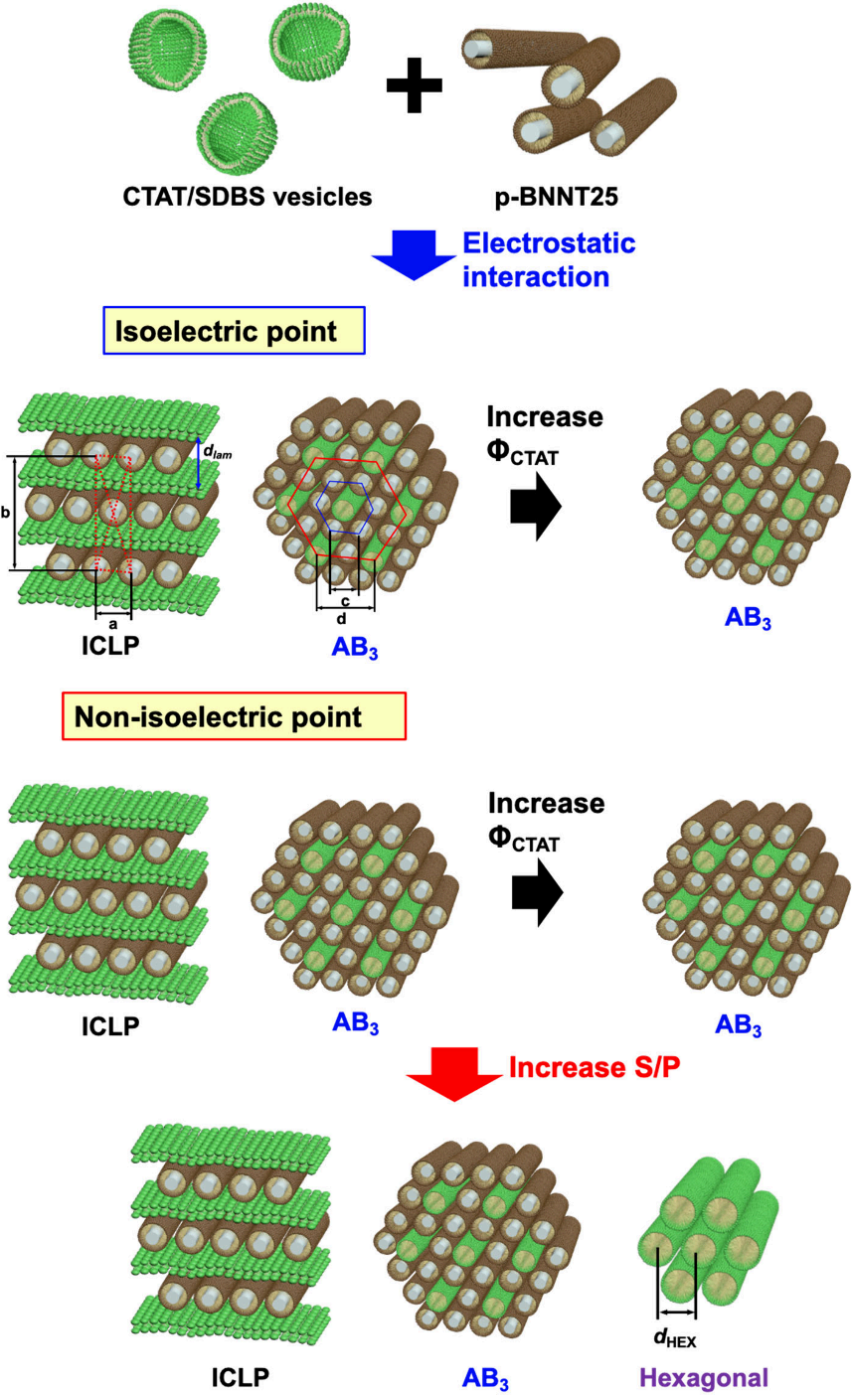
Schematic diagram of two-dimensional binary superlattices of the CTAT/SDBS-BNNT complex induced by electrostatic interactions.

## Conclusion

We have investigated the two-dimensional binary superlattices of CTAT/SDBS-BNNT complexes by electrostatic interactions, which were confirmed by SAXS analysis. Depending on S/P and Φ_CTAT_, the CTAT/SDBS-BNNT complexes self-assembled into the ICLP, AB_3_, and HEX structures. The BNNT surrounded by a cylindrical surfactant micelle (AB_3_) structure and the BNNT inserted between the multilamellar (ICLP) structures of the CTAT/SDBS-BNNT complexes were unique and meaningful, because they have not been observed in other BNNT-amphiphilic molecular complexes. This result, induced by electrostatic interactions, provides a new method to fabricate various structures of BNNTs with improved aggregate properties using surfactants as matrix materials. Furthermore, it is a unique example of inducing a highly ordered self-assembly of one-dimensional (1D) nanoparticles through electrostatic interactions.

## Experimental Section

### Materials

The BNNTs were purchased from the High-Enthalpy Plasma Research Center of Jeonbuk National University and were fabricated using a 60 kW radio frequency inductively coupled plasma system (Iksan, Republic of Korea). Cetyltrimethylammonium tosylate (CTAT), sodium dodecylbenzenesulfonate (SDBS), 4-vinylbenzoic acid (VBA), sodium 4-styrenesulfonate (NaSS), and cetyltrimethylammonium bromide (CTAB) were purchased from Sigma–Aldrich. The VA-044 free radical initiator was purchased from Wako Chemicals. Cetyltrimethylammonium 4-vinylbenzoate (CTVB) amphiphilic surfactants were fabricated in the laboratory using VBA and CTAB. Purified H_2_O (deionized water) was obtained from ELGA PURELAB Option Q.

### Preparation of p-BNNT25

To prepare the p-BNNT25, a functionalized BNNT with a hydrophilic and negatively charged surface, the BNNTs (0.1 wt %) were mixed with a polymerizable cationic surfactant, CTVB (0.5 wt %), and a polymerizable hydrotropic salt, NaSS (25 mol % relative to CTVB), in aqueous solution. The mixed BNNT/CTVB/NaSS solution was sonicated for 1 h using a VCX-750 (Cole-Palmer) sonicator (to exfoliate the BNNT and encapsulate its surface with surfactants), followed by *in situ* free radical copolymerization of the anionic counterions (VB- and SS-) of CTVB and NaSS because of their strongly stable adsorption on the BNNT surface, using a VA-044 free-radical initiator. Subsequently, high-speed centrifugation was performed at 2,502 *g*-force for 30 min to remove impurities and bundled BNNTs. The obtained individually exfoliated BNNTs solution was freeze-dried at −43 °C for 3 days to obtain a powder (that is, p-BNNT25).

### Preparation of CTAT/SDBS Surfactant Vesicles

The cationic surfactant vesicles were obtained through the self-assembly of a mixture of two oppositely charged surfactants, cetyltrimethylammonium tosylate (CTAT, a cationic surfactant) and sodium dodecylbenzenesulfonate (SDBS, an anionic surfactant), with different mole ratios. A mixture of CTAT and SDBS surfactants with Φ_CTAT_ = 0.733, 0.750, 0.765, and 0.779 (where, Φ_CTAT_ is the mole fraction of CTAT) was prepared, and the total surfactant concentration was maintained at 2.5 wt %. The mixtures of CTAT and SDBS surfactants with different ratios were thermally agitated at 60 °C for homogeneous mixing.

### SAXS Measurements

Small angle X-ray scattering (SAXS) measurements were performed on the 4C SAXS beamline of high-resolution X-rays with a wavelength of 0.796 Å (ΔE/E ≈ 2 × 10^–4^) at the Pohang Accelerator Laboratory (PAL) of the Republic of Korea. The sample-to-detector distance (SDD) was fixed at 2 m, and the *q* range was 0.0144 to 0.576 Å. Silver behenate (AgBE) was used as a standard sample to calibrate the *q* range. The beam size was 23 (vertical) × 300 (horizontal) μm.

### SANS Measurements

Small angle neutron scattering (SANS) measurements were performed on two different samples by using distinct instruments. The p-BNNT/NaSS 25 mol % (p-BNNT25) dispersion was measured on the EQ-SANS instrument at the Spallation Neutron Source (SNS) in USA, using neutrons with a minimum wavelength of 2.5 Å and a sample–detector distance (SDD) of 2.5 m, covering a *q* range of 0.007 Å^–1^ to 0.5 Å^–1^. In contrast, the CTAT/SDBS dispersion was measured using the 40 m SANS instrument at HANARO, Korea Atomic Energy Research Institute (KAERI), with a neutron wavelength of 7.49 Å and varying SDDs (1.16, 4.7, and 19.8 m), covering a *q* range of 0.003 Å^–1^ to 0.6 Å^–1^. In both experiments, the scattering intensity was modulated for empty-cell scattering and background, calibrated for detector sensitivity, and processed to obtain 1D averaged scattering intensity on an absolute scale using a software specific to each facility.

### AFM Measurements

The p-BNNT25 in an aqueous solution was spin-cast onto a silicon wafer cleaned by using a piranha solution. Atomic force microscopy (AFM) images were obtained in tapping mode (Bruker, MultiMode 8 model). The coated particles on the Si wafer were burned for 6 h at 450 °C in air to entirely remove the surfactant covered on the BNNTs, which can characterize the length and diameter of the BNNTs.

### UV–vis Measurements

UV–visible spectroscopy was performed by using a Cary 5000 UV–vis–NIR spectrometer (Agilent Technologies). The samples contained a quartz cell with a beam path length of 2 mm at room temperature.

### Zeta Potential Measurements

Zeta potential measurements were obtained by using a Litesizer DLS zeta potential analyzer (Anton Paar). Measurements were recorded in triplicate for each sample.

### TEM Measurements

Transmission electron microscopy (TEM) and energy dispersive X-ray spectroscopy (EDS) measurements were performed using a JEM-ARM200F (JEOL) at the Center for University-wide Research Facilities (CURF) at Jeonbuk National University. The sample was dropped onto a Cu TEM grid at a concentration of 1 mg/mL and dried.

## Supplementary Material



## Notes

Zeta potential analysis of the p-BNNT25 surface depending on NaSS concentration; proton NMR spectrum of p-BNNT25 before and after freeze-drying; SANS form factor analysis of the CTAT/SDBS mixtures; zeta potential analysis of the CTAT/SDBS mixtures; zeta potential analysis of the CTAT/SDBS-BNNT complexes at different Φ_CTAT_; TEM images of the CTAT/SDBS-BNNT (Φ_CTAT_ = 0.750, S/P = 1.0) complex and CTAT/SDBS (Φ_CTAT_ = 0.750) mixture, along with TEM-EDS elemental mapping images of the CTAT/SDBS-BNNT complex; SAXS intensity and lattice parameter of the CTAT/SDBS-BNNT complex (at Φ_CTAT_ = 0.765); comparison of SAXS intensities of ICLP and AB_3_ structures with simulated SAXS patterns of space groups *C*2/*c* (66) and *P*6/*mmm* (191) (PDF)

## Abbreviations

CTAT: cetyltrimethylammonium tosylate
SDBS: sodium dodecylbenzenesulfonate
BNNT: boron nitride nanotube
NaSS: sodium styrenesulfonate
VBA: 4-vinylbenzoic acid
CTAB: cetyltrimethylammonium bromide
CTVB: cetyltrimethylammonium 4-vinylbenzoate
p-BNNT25: negatively charged BNNT
