# Supplementary material for: Two-Dimensional Binary Superlattice of BNNT-Surfactant Vesicle Complex Induced by Electrostatic Interaction
Source: ACS Cent Sci. 2025 May 22;11(6):950–9. doi: 10.1021/acscentsci.5c00548 (PMC12203259; doi:10.1021/acscentsci.5c00548)
Supplement: Supplementary file 1 [file oc5c00548_si_001.pdf]

Supporting information

Two-Dimensional Binary Superlattice of BNNT-Surfactant Vesicle Complex Induced by Electrostatic Interaction

Sang-Woo Jeon[1,2], *Changwoo Do*[3], *Se Youn Moon*[1,4,5,6], *Tae-Hwan Kim*[1,4,5,6,7,8*]

[1]Department of Applied Plasma & Quantum Beam Engineering, Jeonbuk National University, Jeonju 54896, Republic of Korea

[2]Neutron Science Division, Korea Atomic Energy Research Institute, Daejeon, 34057, Republic of Korea

[3]Biology and Soft Matter Division, Neutron Sciences Directorate, Oak Ridge National Laboratory, Oak Ridge, TN 37831, USA

[4]Research Center for Advanced Nuclear Interdisciplinary Technology, Jeonbuk National University, Jeonju 54896, Republic of Korea

[5]Department of Quantum System Engineering, Jeonbuk National University, Jeonju 54896, Republic of Korea

[6]High-Enthalphy Plasma Research Center, Jeonbuk National University, Wanju-gun, 55317, Republic of Korea.

[7]Department of Electronic Engineering, Jeonbuk National University, Jeonju 54896, Republic of Korea

[8]Department of JBNU-KIST Convergence, Jeonbuk National University, Jeonju 54896, Republic of Korea

*Corresponding author: taehwan@jbnu.ac.kr

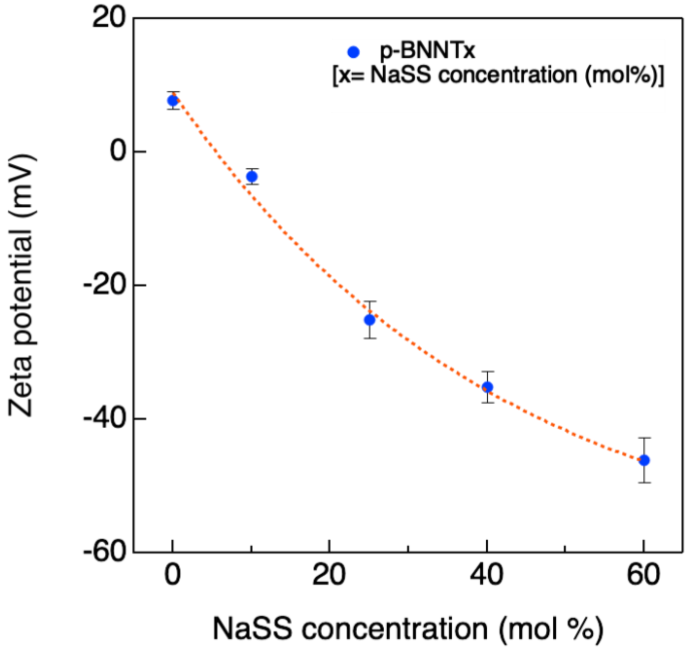

Figure S1. The zeta potential of the p-BNNT surface depending on NaSS concentration (mol%).

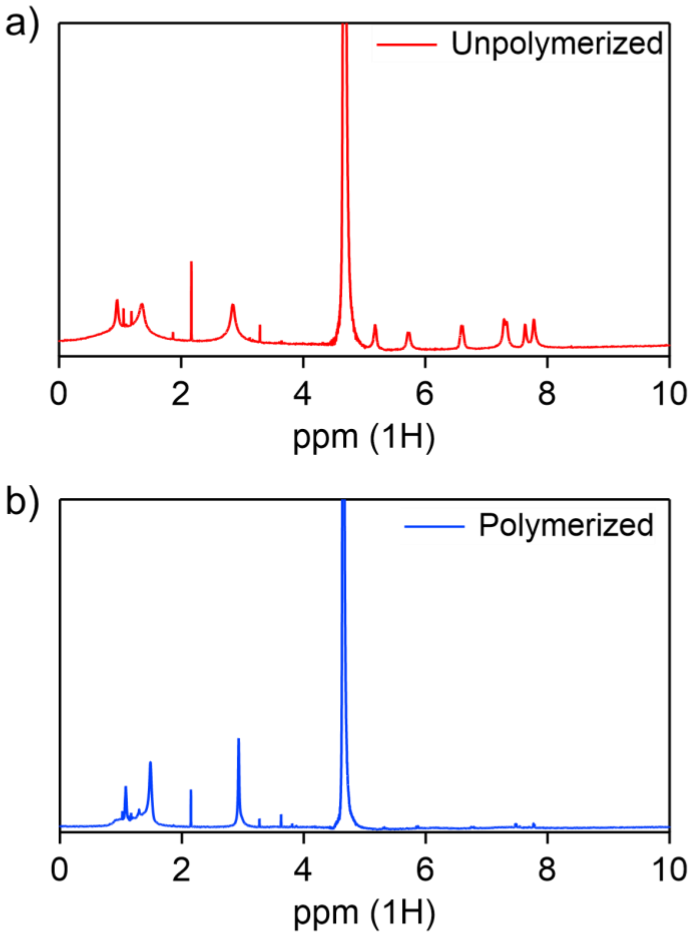

Figure S2. Proton NMR spectrum of a) unpolymerized and b) polymerized permanently exfoliated and negatively charged BNNTs.

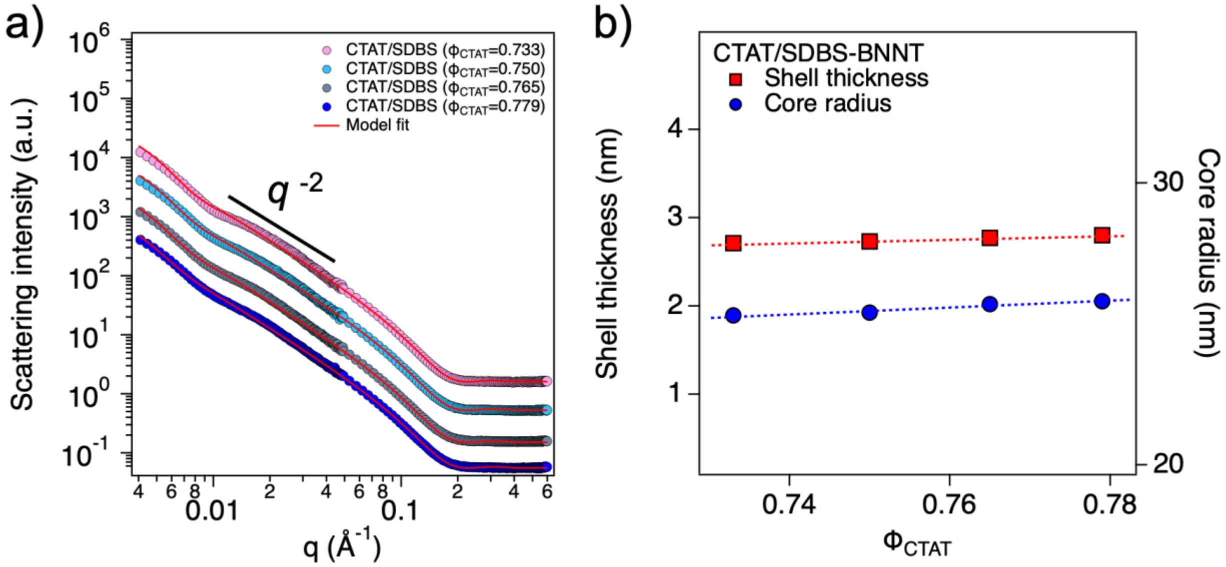

Figure S3. a) SANS form factor analysis of the CTAT/SDBS vesicles. b) Shell thickness and core radius of the CTAT/SDBS vesicle.

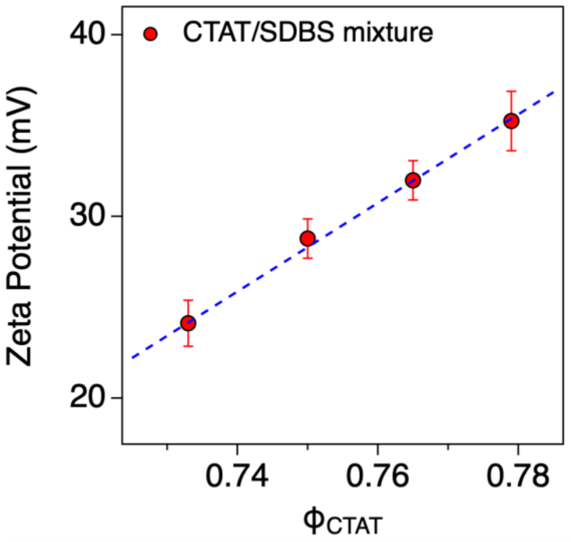

Figure S4. Surface charge densities of the CTAT/SDBS vesicles at different Φ_{CTAT}.

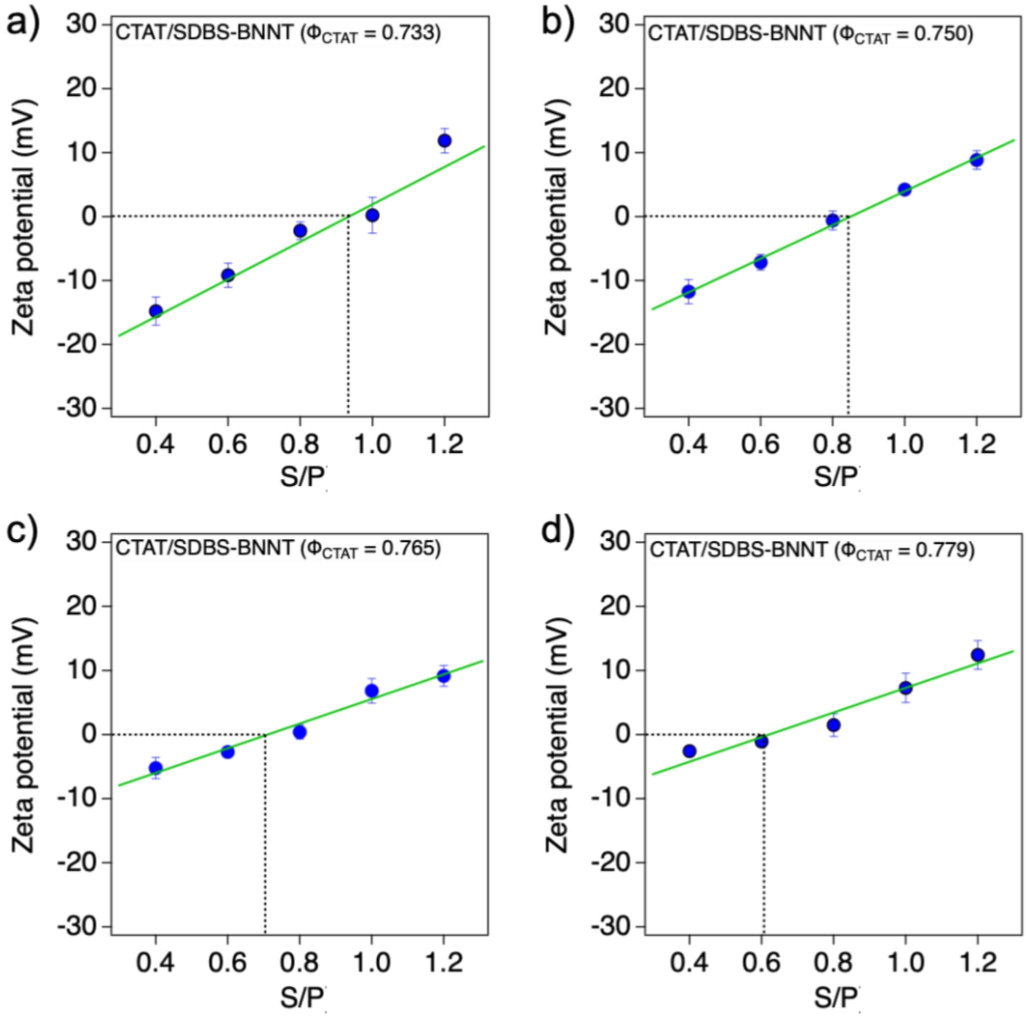

Figure S5. Surface charge density of the CTAT/SDBS-BNNT complexes at Φ_{CTAT} is a) 0.733, b) 0.750, c) 0.765, d) 0.779.

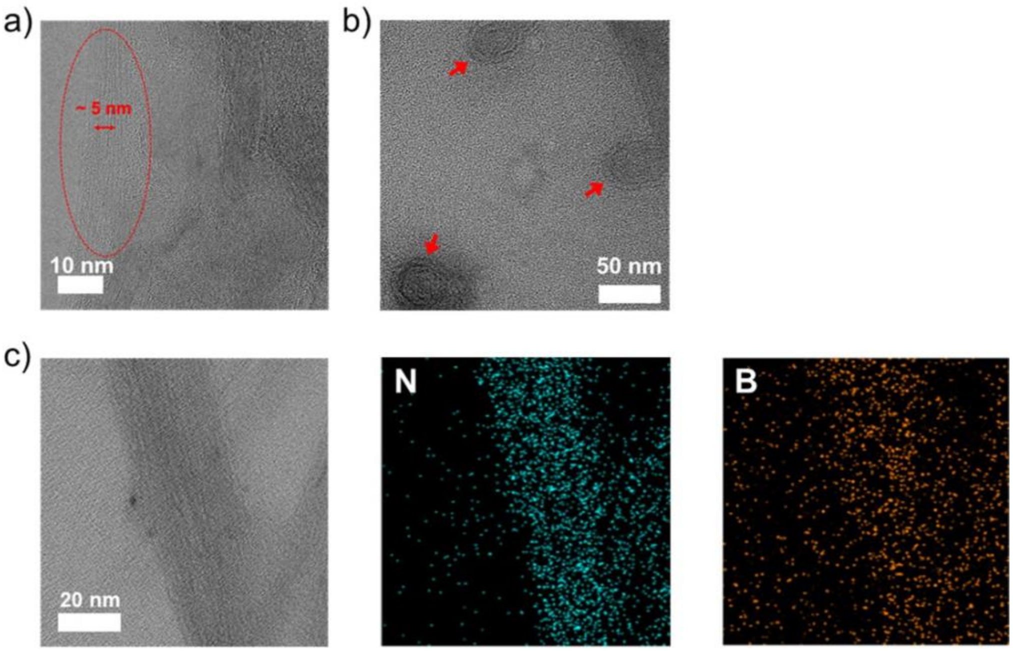

Figure S6. TEM images of a) CTAT/SDBS-BNNT complex (Φ_{CTAT} = 0.750, S/P = 1.0) and b) CTAT/SDBS mixture (Φ_{CTAT} = 0.750, where red arrows indicate the vesicles). c) TEM-EDS elemental mapping images of CTAT/SDBS-BNNT (Φ_{CTAT} = 0.750, S/P = 1.0) complex, showing distributions of d) nitrogen (N) and e) boron (B) atoms.

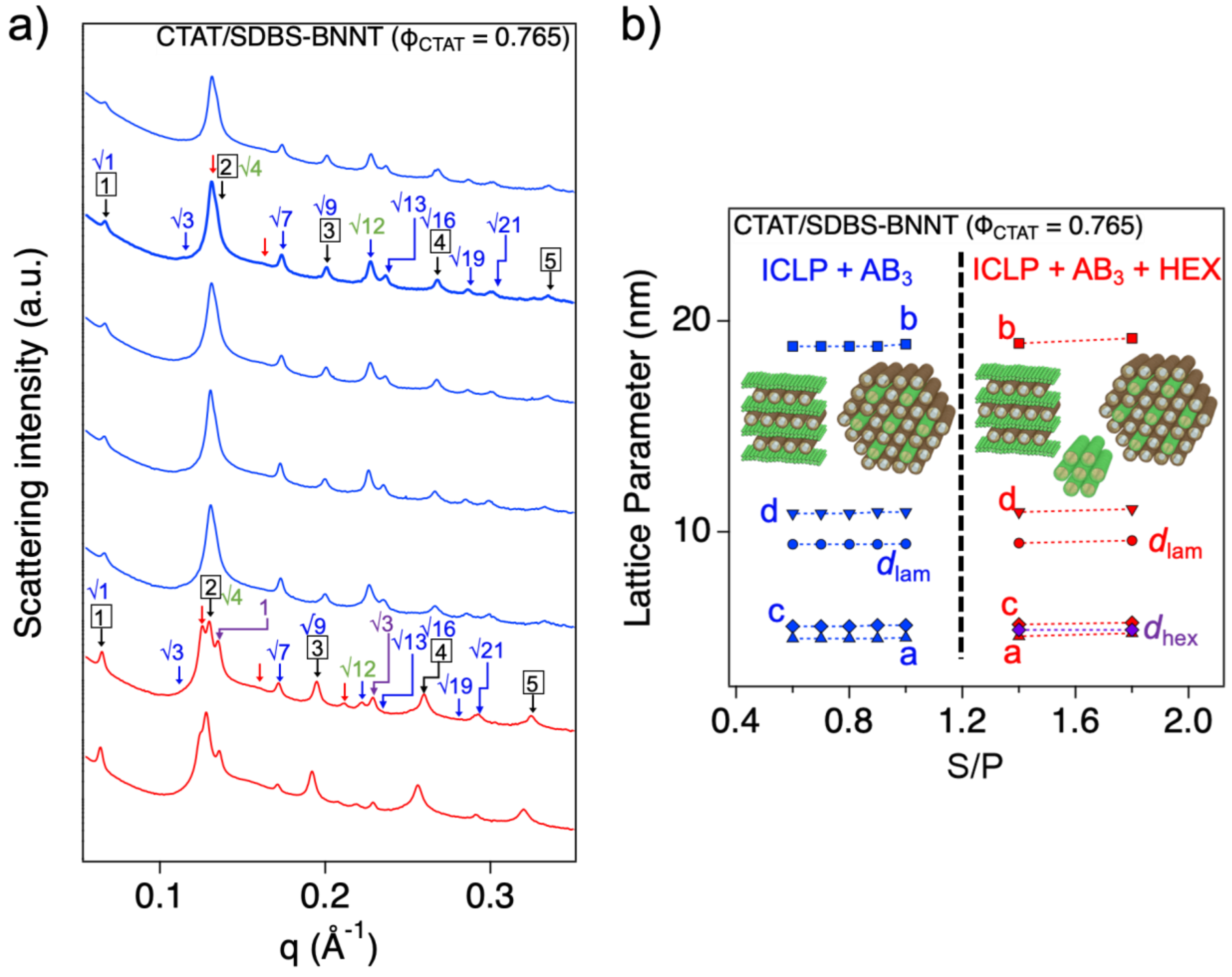

Figure S7. a) SAXS intensity and b) lattice parameter of the CTAT/SDBS-BNNT ($\Phi_{CTAT} = 0.765$) complex.

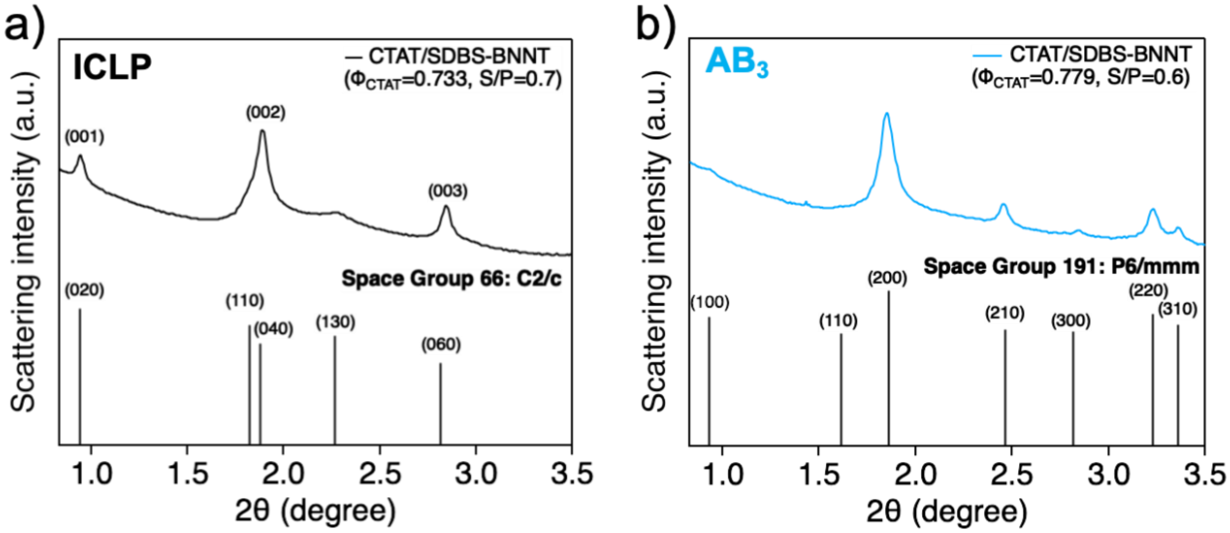

Figure S8. SAXS intensities of (a) ICLP and (b) AB_3 structure compared with simulated SAXS pattern of space group C2/c (66) (a = 50.10 Å, b = 188.20 Å. c = 0 Å; α = β = γ = 90 °) and space group P6/mmm (191) (a = b = 108.60 Å. c = 0 Å; α = β = 90 °, γ = 120 °).
